# Crystal structure of *N*-{*N*-[*N*-acetyl-(*S*)-leuc­yl]-(*S*)-leuc­yl}norleucinal (ALLN), an inhibitor of proteasome

**DOI:** 10.1107/S2056989015002091

**Published:** 2015-02-07

**Authors:** Andrzej Czerwinski, Channa Basava, Miroslawa Dauter, Zbigniew Dauter

**Affiliations:** a Peptides International, Inc., 11621 Electron Drive, Louisville, KY 40299, USA; b Leidos Biomedical Research Inc., Basic Science Program, Argonne National Laboratory, Argonne, IL 60439, USA; c Synchrotron Radiation Research Section, MCL, National Cancer Institute, Argonne National Laboratory, Argonne, IL 60439, USA

**Keywords:** crystal structure, proteasome inhib­itor, hydrogen bonding, anti­parallel β-sheet.

## Abstract

The title compound, C_20_H_37_N_3_O_4_, also known by the acronym ALLN, is a tripeptidic inhibitor of the proteolytic activity of the proteasomes, enzyme complexes implicated in several neurodegenerative diseases and other disorders, including cancer. The crystal structure of ALLN, solved from synchrotron radiation diffraction data, revealed the mol­ecules in extended conformation of the backbone and engaging all peptide N and O atoms in inter­molecular hydrogen bonds forming an infinite anti­parallel β-sheet.

## Abstract 2

The crystal structure of ALLN, the tripeptidic inhibitor of proteasomes, is solved from synchrotron diffraction data. An infinite β-sheet extended through the crystal is formed by symmetry-related oligopeptide mol­ecules in extended conformation.

## Chemical context   

Proteasomes are high-mol­ecular-mass multicatalytic enzyme complexes localized in the nucleus and cytosol of all eukaryotic cells. As a part of the ubiquitin–proteasome pathway, the complex executes a remarkable set of functions, ranging from the complete destruction of abnormal and misfolded proteins to the specific proteolytic activation of crucial signaling mol­ecules (Adams, 2003 ▸; Groll & Potts, 2011 ▸). The ubiquitin–proteasome pathway has been implicated in several forms of malignancy, in the pathogenesis of some autoimmune disorders, the aging process related cardiac dysfunction, diabetic complications, and neurodegenerative diseases (*e.g.* Alzheimer’s, Parkinson’s, Huntington’s) (Dahlmann, 2007 ▸; Paul, 2008 ▸; Jankowska *et al.*, 2013 ▸). Therefore, study of proteasome functions and the design and development of proteasome inhibitors is being pursued in many laboratories (Bennett & Kirk, 2008 ▸). A great amount of effort has been expended to explore proteasome inhibition as a novel targeted approach in cancer therapy. The first success came with FDA approval of Bortezomid for the treatment of multiple myeloma (Kane *et al.*, 2006 ▸; Goldberg, 2012 ▸). Since then, numerous compounds have been reported to inhibit the components of the ubiquitin–proteasome system, and several new drug candidates undergoing clinical trials have emerged (Genin *et al.*, 2010 ▸; Tsukamoto & Yokosawa, 2010 ▸; Frankland-Searby & Bhaumik, 2012 ▸; Jankowska *et al.*, 2013 ▸). Peptide aldehydes were the first inhibitors designed to target the proteasome, and are still the most commonly used and best characterized group of such inhibitors (Kisselev *et al.*, 2012 ▸). A notable one among them, Ac-Leu-Leu-Nle-H (ALLN, MG101), is also a potent inhibitor of nonproteasomal cysteine protease calpain I (Pietsch *et al.*, 2010 ▸). ALLN, a cell-permeable tripeptide aldehyde reversible inhibitor of chymotripsin-like proteolytic activity of the proteasomes, was the first to be crystallized in a complex with an eukaryotic proteasome (Groll *et al.*, 1997 ▸). Crystallographic analysis of the complex at 2.4 Å resolution revealed a structural organization of the proteasome and how the inhibitor binds to its active site. ALLN, as well as other peptide aldehydes, do it *via* reversible hemiacetal formation with the involvement of N-terminal threonine hy­droxy group in the proteasome β-subunits (Borissenko & Groll, 2007 ▸). The aldehyde structure derived from the crystal complex coordinates was used in mol­ecular modeling of inhibitor-proteasome inter­actions (Zhang *et al.*, 2009 ▸). High resolution structural data from this study may provide better accuracy in future modeling of the inhibitor inter­actions with proteasome and other potential intra­cellular targets.
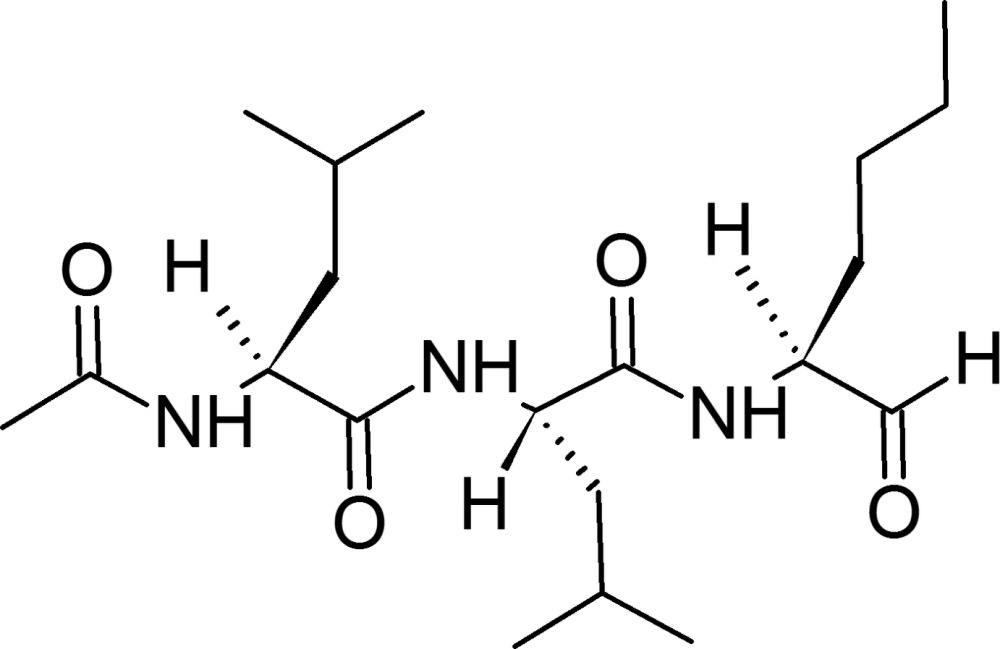


## Structural commentary   

**Figure 1:**
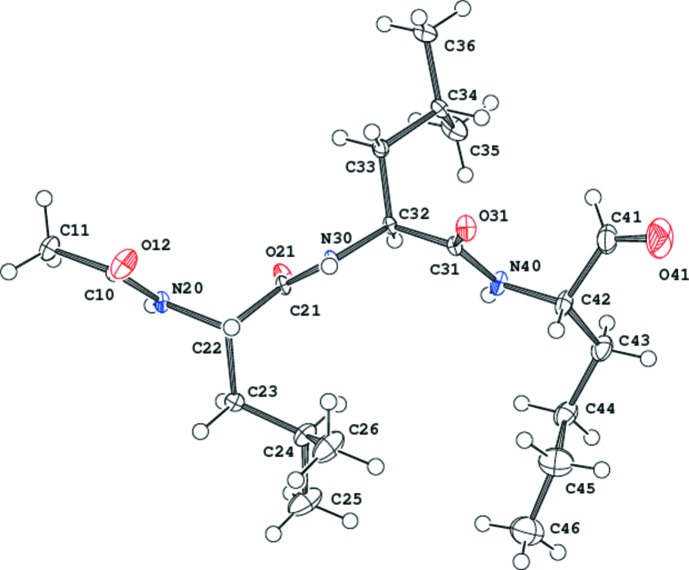
The mol­ecule of ALLN, showing the atom-labelling scheme. Displacement ellipsoids are drawn at the 50% probability level.

We report here the crystal structure of ALLN refined against 0.65 Å resolution diffraction data measured with synchrotron radiation. The mol­ecule adopts an extended conformation of the backbone chain (Fig. 1 ▸) with the ϕ,ψ-torsion angles residing in the β region of the Ramachandran plot (Ramakrishnan & Ramachandran, 1965 ▸). All three consecutive peptide residues are in *trans* conformation and their ω angles are −179.42 (9), 173.77 (8), and 177.72 (10)°. The side chains of the two leucine and one norleucine residues have unstrained conformations, and do not deviate by more than 7° from either *trans* or *gauche* rotamers along the consecutive C—C bonds.

## Supra­molecular features   

**Figure 2:**
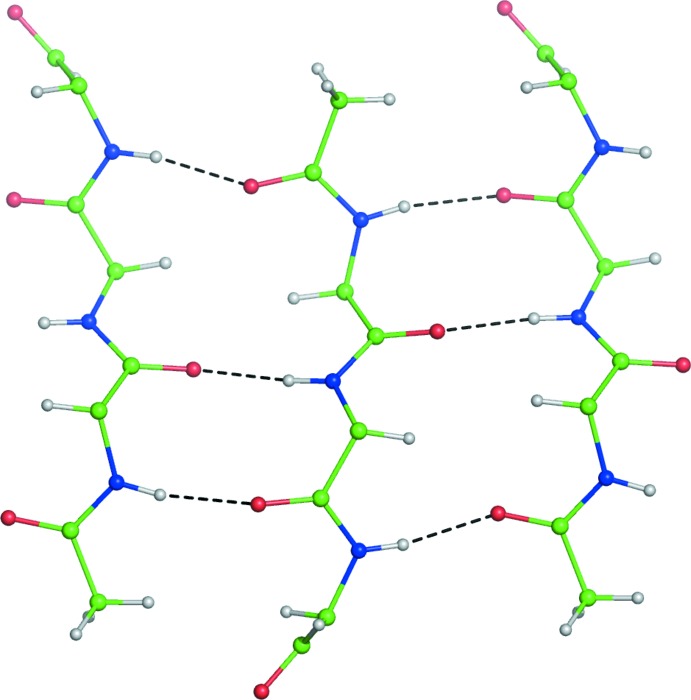
Backbones of three neighboring mol­ecules of ALLN, forming a fragment of an anti­parallel β-sheet extending through the crystal. The amino acid side chains are not shown for clarity.

**Figure 3:**
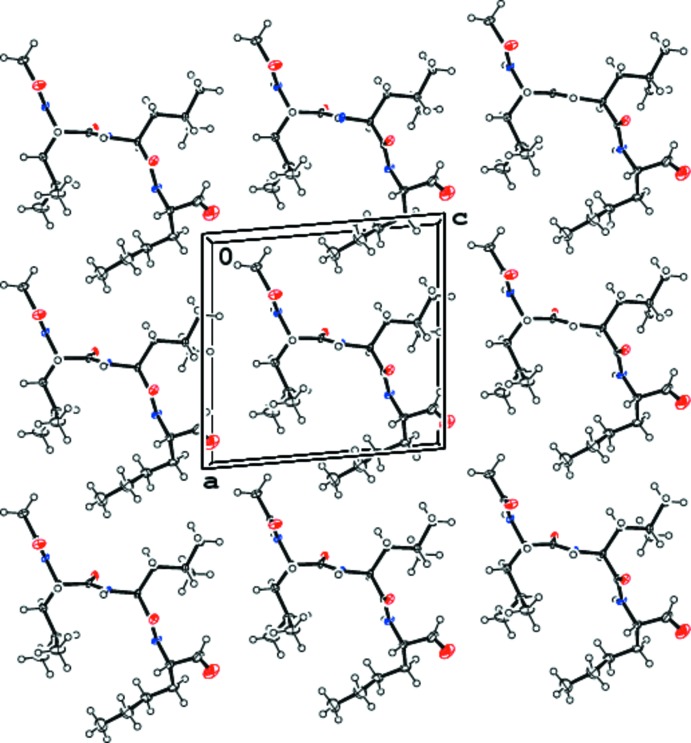
Arrangement of ALLN mol­ecules in the *ac* plane of the crystal, inter­acting through their aliphatic side chains.

All of the peptide ALLN N and O atoms are engaged in inter­molecular hydrogen bonds (Table 1 ▸) between mol­ecules related by the crystallographic 2_1_ axis, forming an infinite anti­parallel β-sheet throughout the crystal (Fig. 2 ▸). The inter­actions between the sheets are mainly by the hydro­phobic contacts of the aliphatic amino acid side chains. The arrangement of ALLN molecules in the *ac* plane, interacting through their aliphatic side chains, is illustrated in Fig. 3 ▸.

## Synthesis and crystallization   

The title aldehyde was prepared according to the general synthetic procedure reported by Schaschke *et al.* (1996 ▸), and a 45% overall yield was obtained. The product was crystallized from aceto­nitrile.

## Refinement details   

Crystal data, data collection and structure refinement details are summarized in Table 2 ▸. A needle-like crystal elongated in the *a* direction was selected, picked up in the rayon loop and then quickly cryo-cooled in a stream of cold nitro­gen gas at the single-axis goniostat of the SER-CAT synchrotron station ID19 at the Advanced Photon Source, Argonne National Laboratory, USA. Diffraction images were collected with the use of MAR300 CCD detector in two passes differing in the effective exposure and resolution limits in order to adequately measure the weakest high-resolution reflections, as well as the strongest low-angle reflections without overloading detector pixels. All 38117 measured intensities from both passes were integrated, scaled and merged by *HKL-2000* (Otwinowski & Minor, 1997 ▸) into the set of 4561 unique reflections with the overall *R*_merge_ factor of 0.049. The data set is rather strong, with the *I*/σ(*I*) ratio equal to 25 at the highest resolution of 0.65 Å. H atoms were located in a difference synthesis and refined as riding on their parent atoms in geometrically idealized positions. Because of the short wavelength of synchrotron radiation, all Friedel mates were averaged during data processing. The chirality of the mol­ecule was deduced from the known chiral centres in the substrates used in chemical synthesis.

## Supplementary Material

Crystal structure: contains datablock(s) I. DOI: 10.1107/S2056989015002091/gk2625sup1.cif

Structure factors: contains datablock(s) I. DOI: 10.1107/S2056989015002091/gk2625Isup2.hkl

CCDC reference: 1046561

Additional supporting information:  crystallographic information; 3D view; checkCIF report

## Figures and Tables

**Table 1:** Hydrogen-bond geometry (, )

*D*H*A*	*D*H	H*A*	*D* *A*	*D*H*A*
N20H201O31^i^	0.88	2.05	2.897(3)	161
N30H301O21^ii^	0.88	1.99	2.863(3)	171
N40H401O12^i^	0.88	1.96	2.827(3)	169

Symmetry codes: (i) 
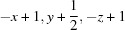
; (ii) 
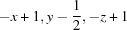
.

**Table 2:** Experimental details

Crystal data	
Chemical formula	C_20_H_37_N_3_O_4_
*M*_r_	383.59
Crystal system, space group	Monoclinic, *P*2_1_
Temperature (K)	100
*a*, *b*, *c* ()	10.85(1), 9.510(9), 11.200(11)
()	94.85(2)
*V* (^3^)	1152(2)
*Z*	2
Radiation type	Synchrotron, = 0.6199
(mm^1^)	0.09
Crystal size (mm)	0.30 0.05 0.02

Data collection	
Diffractometer	MAR300 CCD
Absorption correction	Multi-scan (*SCALEPACK*; Otwinowski *et al.*, 2003 ▸)
*T*_min_, *T*_max_	0.974, 0.999
No. of measured, independent and observed [*I* > 2(*I*)] reflections	4561, 4561, 4492
*R*_int_	0.049
(sin /)_max_ (^1^)	0.767

Refinement	
*R*[*F* ^2^ > 2(*F* ^2^)], *wR*(*F* ^2^), *S*	0.041, 0.115, 1.07
No. of reflections	4561
No. of parameters	244
No. of restraints	1
H-atom treatment	H-atom parameters constrained
_max_, _min_ (e ^3^)	0.44, 0.29

Computer programs: *HKL-2000* (Otwinowski Minor, 1997 ▸), *SHELXD* and *SHELXL97* (Sheldrick, 2008 ▸), *ORTEP-3* for Windwows (Farrugia, 2012 ▸) and *pyMOL* (DeLano, 2002 ▸).

## supplementary crystallographic information

### Crystal data

**Table:**

C_20_H_37_N_3_O_4_	*F*(000) = 460
*M**_r_* = 383.59	*D*_x_ = 1.110 Mg m^−^^3^
Monoclinic, *P*2_1_	Synchrotron radiation, λ = 0.6199 Å
*a* = 10.85 (1) Å	θ = 1.5–28.4°
*b* = 9.510 (9) Å	µ = 0.09 mm^−^^1^
*c* = 11.200 (11) Å	*T* = 100 K
β = 94.85 (2)°	Needle, colourless
*V* = 1152 (2) Å^3^	0.30 × 0.05 × 0.02 mm
*Z* = 2	

### Data collection

**Table:**

MAR300 CCD diffractometer	4561 independent reflections
Radiation source: SER-CAT 22ID synchrotron beamline, APS, USA	4492 reflections with *I* > 2σ(*I*)
Si111 double crystal monochromator	*R*_int_ = 0.049
ω scans	θ_max_ = 28.4°, θ_min_ = 1.5°
Absorption correction: multi-scan (*SCALEPACK*; Otwinowski *et al.*, 2003)	*h* = 0→16
*T*_min_ = 0.974, *T*_max_ = 0.999	*k* = 0→14
4561 measured reflections	*l* = −17→17

### Refinement

**Table:**

Refinement on *F*^2^	Primary atom site location: structure-invariant direct methods
Least-squares matrix: full	Secondary atom site location: difference Fourier map
*R*[*F*^2^ > 2σ(*F*^2^)] = 0.041	Hydrogen site location: inferred from neighbouring sites
*wR*(*F*^2^) = 0.115	H-atom parameters constrained
*S* = 1.07	*w* = 1/[σ^2^(*F*_o_^2^) + (0.081*P*)^2^ + 0.1533*P*] where *P* = (*F*_o_^2^ + 2*F*_c_^2^)/3
4561 reflections	(Δ/σ)_max_ < 0.001
244 parameters	Δρ_max_ = 0.44 e Å^−^^3^
1 restraint	Δρ_min_ = −0.29 e Å^−^^3^

### Special details

**Table:**

Experimental. diffraction data were measured at the station 22ID of the APS synchrotron by rotation method a in three sweeps of different exposure and all data were scaled and merged into one data set
Geometry. All e.s.d.'s (except the e.s.d. in the dihedral angle between two l.s. planes) are estimated using the full covariance matrix. The cell e.s.d.'s are taken into account individually in the estimation of e.s.d.'s in distances, angles and torsion angles; correlations between e.s.d.'s in cell parameters are only used when they are defined by crystal symmetry. An approximate (isotropic) treatment of cell e.s.d.'s is used for estimating e.s.d.'s involving l.s. planes.
Refinement. Refinement of *F*^2^ against all reflections. The weighted *R*-factor *wR* and goodness of fit *S* are based on *F*^2^, conventional *R*-factors *R* are based on *F*, with *F* set to zero for negative *F*^2^. The threshold expression of *F*^2^ > σ(*F*^2^) is used only for calculating *R*-factors(gt) *etc*. and is not relevant to the choice of reflections for refinement. *R*-factors based on *F*^2^ are statistically about twice as large as those based on *F*, and *R*- factors based on all data will be even larger.

### Fractional atomic coordinates and isotropic or equivalent isotropic displacement parameters (Å^2^)

**Table:**

	*x*	*y*	*z*	*U*_iso_*/*U*_eq_	
C10	0.26159 (10)	0.47871 (12)	0.27033 (9)	0.01223 (18)	
C11	0.14253 (11)	0.53384 (15)	0.20793 (12)	0.0195 (2)	
H101	0.1486	0.6359	0.1981	0.029*	
H102	0.0737	0.5120	0.2562	0.029*	
H103	0.1280	0.4893	0.1291	0.029*	
O12	0.27570 (9)	0.35222 (10)	0.29248 (11)	0.02159 (19)	
N20	0.34966 (8)	0.57472 (10)	0.29951 (8)	0.01103 (15)	
H201	0.3336	0.6633	0.2818	0.013*	
C21	0.48033 (9)	0.60494 (11)	0.48414 (8)	0.00972 (16)	
O21	0.46004 (9)	0.73127 (9)	0.49827 (7)	0.01513 (16)	
C22	0.47046 (9)	0.54014 (11)	0.35897 (8)	0.00941 (16)	
H221	0.4804	0.4358	0.3648	0.011*	
C23	0.57151 (10)	0.60292 (12)	0.28663 (10)	0.01324 (18)	
H231	0.5686	0.5542	0.2083	0.016*	
H232	0.5515	0.7031	0.2705	0.016*	
C24	0.70443 (11)	0.59414 (15)	0.34574 (12)	0.0197 (2)	
H241	0.7063	0.6398	0.4263	0.024*	
C25	0.79082 (15)	0.6765 (2)	0.2697 (2)	0.0362 (4)	
H251	0.7606	0.7731	0.2589	0.054*	
H252	0.7927	0.6312	0.1913	0.054*	
H253	0.8744	0.6777	0.3103	0.054*	
C26	0.75042 (14)	0.44426 (18)	0.36297 (19)	0.0322 (3)	
H261	0.6947	0.3922	0.4114	0.048*	
H262	0.8339	0.4450	0.4040	0.048*	
H263	0.7524	0.3987	0.2847	0.048*	
N30	0.51541 (8)	0.51775 (10)	0.57466 (8)	0.01027 (15)	
H301	0.5237	0.4274	0.5606	0.012*	
C31	0.65117 (9)	0.49218 (11)	0.75420 (9)	0.01002 (17)	
O31	0.64628 (8)	0.36532 (9)	0.77767 (8)	0.01517 (16)	
C32	0.53994 (9)	0.57155 (11)	0.69646 (8)	0.00945 (16)	
H321	0.5607	0.6738	0.6928	0.011*	
C33	0.42768 (10)	0.55287 (12)	0.76899 (9)	0.01255 (18)	
H331	0.4034	0.4525	0.7665	0.015*	
H332	0.3578	0.6075	0.7299	0.015*	
C34	0.44800 (10)	0.59874 (13)	0.90039 (9)	0.01353 (18)	
H341	0.5167	0.5408	0.9400	0.016*	
C35	0.48372 (15)	0.75302 (16)	0.91469 (12)	0.0239 (3)	
H351	0.4956	0.7765	1.0001	0.036*	
H352	0.4178	0.8118	0.8759	0.036*	
H353	0.5608	0.7701	0.8773	0.036*	
C36	0.33127 (12)	0.56946 (18)	0.96323 (11)	0.0233 (3)	
H361	0.3442	0.5988	1.0472	0.035*	
H362	0.3128	0.4686	0.9594	0.035*	
H363	0.2618	0.6221	0.9235	0.035*	
N40	0.75393 (9)	0.56824 (11)	0.77879 (9)	0.01460 (17)	
H401	0.7556	0.6578	0.7593	0.018*	
C41	0.83380 (14)	0.45232 (19)	0.96234 (14)	0.0283 (3)	
H411	0.7627	0.4898	0.9949	0.034*	
O41	0.89574 (15)	0.3697 (2)	1.02126 (16)	0.0518 (5)	
C42	0.86236 (11)	0.50104 (14)	0.83793 (12)	0.0187 (2)	
H421	0.8849	0.4177	0.7898	0.022*	
C43	0.97094 (12)	0.60363 (17)	0.84929 (13)	0.0235 (2)	
H431	1.0416	0.5583	0.8960	0.028*	
H432	0.9471	0.6874	0.8945	0.028*	
C44	1.01242 (12)	0.65110 (17)	0.72874 (14)	0.0244 (3)	
H441	0.9426	0.6996	0.6833	0.029*	
H442	1.0805	0.7200	0.7432	0.029*	
C45	1.05617 (19)	0.5319 (2)	0.65273 (18)	0.0373 (4)	
H451	0.9855	0.4686	0.6307	0.045*	
H452	1.1196	0.4770	0.7013	0.045*	
C46	1.1102 (2)	0.5807 (3)	0.53884 (19)	0.0458 (5)	
H461	1.1363	0.4987	0.4943	0.069*	
H462	1.1818	0.6414	0.5597	0.069*	
H463	1.0474	0.6333	0.4891	0.069*	

### Atomic displacement parameters (Å^2^)

**Table:**

	*U*^11^	*U*^22^	*U*^33^	*U*^12^	*U*^13^	*U*^23^
C10	0.0124 (4)	0.0105 (4)	0.0138 (4)	0.0002 (3)	0.0009 (3)	−0.0023 (3)
C11	0.0134 (4)	0.0200 (6)	0.0243 (5)	0.0018 (4)	−0.0028 (4)	0.0005 (4)
O12	0.0181 (4)	0.0080 (4)	0.0378 (5)	−0.0016 (3)	−0.0023 (3)	−0.0013 (4)
N20	0.0141 (3)	0.0063 (3)	0.0121 (3)	0.0006 (3)	−0.0030 (3)	0.0008 (3)
C21	0.0143 (4)	0.0062 (4)	0.0083 (4)	0.0003 (3)	−0.0016 (3)	−0.0008 (3)
O21	0.0277 (4)	0.0051 (3)	0.0119 (3)	0.0031 (3)	−0.0024 (3)	−0.0008 (3)
C22	0.0135 (4)	0.0060 (4)	0.0082 (3)	0.0008 (3)	−0.0019 (3)	−0.0008 (3)
C23	0.0150 (4)	0.0110 (4)	0.0139 (4)	0.0012 (3)	0.0020 (3)	−0.0001 (3)
C24	0.0141 (4)	0.0181 (5)	0.0267 (5)	0.0019 (4)	0.0008 (4)	−0.0001 (4)
C25	0.0208 (6)	0.0296 (8)	0.0591 (11)	−0.0023 (6)	0.0089 (6)	0.0118 (8)
C26	0.0195 (5)	0.0219 (7)	0.0548 (10)	0.0055 (5)	0.0012 (6)	0.0079 (7)
N30	0.0172 (4)	0.0056 (3)	0.0075 (3)	0.0006 (3)	−0.0020 (3)	−0.0005 (3)
C31	0.0132 (4)	0.0072 (4)	0.0092 (3)	0.0003 (3)	−0.0015 (3)	−0.0003 (3)
O31	0.0204 (4)	0.0062 (3)	0.0180 (3)	−0.0007 (3)	−0.0035 (3)	0.0011 (3)
C32	0.0137 (4)	0.0065 (4)	0.0077 (3)	0.0000 (3)	−0.0015 (3)	−0.0008 (3)
C33	0.0133 (4)	0.0132 (4)	0.0109 (4)	−0.0011 (3)	−0.0002 (3)	−0.0013 (3)
C34	0.0167 (4)	0.0143 (5)	0.0096 (4)	0.0008 (4)	0.0013 (3)	−0.0005 (3)
C35	0.0375 (7)	0.0162 (6)	0.0185 (5)	−0.0037 (5)	0.0052 (5)	−0.0075 (4)
C36	0.0211 (5)	0.0331 (7)	0.0164 (5)	0.0007 (5)	0.0065 (4)	0.0021 (5)
N40	0.0135 (4)	0.0083 (4)	0.0209 (4)	−0.0013 (3)	−0.0051 (3)	0.0030 (3)
C41	0.0235 (6)	0.0324 (8)	0.0273 (6)	−0.0049 (5)	−0.0078 (5)	0.0109 (6)
O41	0.0423 (7)	0.0570 (11)	0.0533 (9)	0.0014 (7)	−0.0120 (6)	0.0360 (8)
C42	0.0142 (4)	0.0163 (5)	0.0242 (5)	−0.0006 (4)	−0.0061 (4)	0.0056 (4)
C43	0.0170 (5)	0.0249 (6)	0.0276 (6)	−0.0069 (5)	−0.0039 (4)	0.0006 (5)
C44	0.0181 (5)	0.0226 (6)	0.0323 (6)	−0.0030 (5)	0.0002 (4)	0.0042 (5)
C45	0.0417 (9)	0.0321 (9)	0.0394 (8)	−0.0022 (7)	0.0114 (7)	−0.0022 (7)
C46	0.0419 (9)	0.0611 (15)	0.0356 (8)	−0.0080 (10)	0.0094 (7)	−0.0023 (9)

### Geometric parameters (Å, º)

**Table:**

C10—O12	1.2351 (19)	C33—C34	1.533 (2)
C10—N20	1.3420 (17)	C33—H331	0.9900
C10—C11	1.5097 (19)	C33—H332	0.9900
C11—H101	0.9800	C34—C35	1.522 (2)
C11—H102	0.9800	C34—C36	1.526 (2)
C11—H103	0.9800	C34—H341	1.0000
N20—C22	1.4567 (17)	C35—H351	0.9800
N20—H201	0.8800	C35—H352	0.9800
C21—O21	1.2340 (18)	C35—H353	0.9800
C21—N30	1.3398 (16)	C36—H361	0.9800
C21—C22	1.5268 (19)	C36—H362	0.9800
C22—C23	1.5380 (18)	C36—H363	0.9800
C22—H221	1.0000	N40—C42	1.4489 (17)
C23—C24	1.537 (2)	N40—H401	0.8800
C23—H231	0.9900	C41—O41	1.196 (2)
C23—H232	0.9900	C41—C42	1.525 (2)
C24—C26	1.517 (3)	C41—H411	0.9500
C24—C25	1.534 (2)	C42—C43	1.527 (2)
C24—H241	1.0000	C42—H421	1.0000
C25—H251	0.9800	C43—C44	1.527 (2)
C25—H252	0.9800	C43—H431	0.9900
C25—H253	0.9800	C43—H432	0.9900
C26—H261	0.9800	C44—C45	1.517 (3)
C26—H262	0.9800	C44—H441	0.9900
C26—H263	0.9800	C44—H442	0.9900
N30—C32	1.4602 (18)	C45—C46	1.521 (3)
N30—H301	0.8800	C45—H451	0.9900
C31—O31	1.2369 (18)	C45—H452	0.9900
C31—N40	1.3380 (16)	C46—H461	0.9800
C31—C32	1.5207 (17)	C46—H462	0.9800
C32—C33	1.5305 (18)	C46—H463	0.9800
C32—H321	1.0000		
			
O12—C10—N20	122.69 (12)	C34—C33—H331	108.6
O12—C10—C11	121.19 (11)	C32—C33—H332	108.6
N20—C10—C11	116.12 (12)	C34—C33—H332	108.6
C10—C11—H101	109.5	H331—C33—H332	107.5
C10—C11—H102	109.5	C35—C34—C36	109.95 (11)
H101—C11—H102	109.5	C35—C34—C33	112.95 (10)
C10—C11—H103	109.5	C36—C34—C33	109.47 (10)
H101—C11—H103	109.5	C35—C34—H341	108.1
H102—C11—H103	109.5	C36—C34—H341	108.1
C10—N20—C22	123.53 (11)	C33—C34—H341	108.1
C10—N20—H201	118.2	C34—C35—H351	109.5
C22—N20—H201	118.2	C34—C35—H352	109.5
O21—C21—N30	123.22 (11)	H351—C35—H352	109.5
O21—C21—C22	120.75 (9)	C34—C35—H353	109.5
N30—C21—C22	116.00 (11)	H351—C35—H353	109.5
N20—C22—C21	108.61 (9)	H352—C35—H353	109.5
N20—C22—C23	108.99 (10)	C34—C36—H361	109.5
C21—C22—C23	109.22 (10)	C34—C36—H362	109.5
N20—C22—H221	110.0	H361—C36—H362	109.5
C21—C22—H221	110.0	C34—C36—H363	109.5
C23—C22—H221	110.0	H361—C36—H363	109.5
C24—C23—C22	115.88 (11)	H362—C36—H363	109.5
C24—C23—H231	108.3	C31—N40—C42	119.07 (12)
C22—C23—H231	108.3	C31—N40—H401	120.5
C24—C23—H232	108.3	C42—N40—H401	120.5
C22—C23—H232	108.3	O41—C41—C42	123.77 (18)
H231—C23—H232	107.4	O41—C41—H411	118.1
C26—C24—C25	109.90 (13)	C42—C41—H411	118.1
C26—C24—C23	113.10 (11)	N40—C42—C41	109.39 (12)
C25—C24—C23	109.16 (13)	N40—C42—C43	110.32 (13)
C26—C24—H241	108.2	C41—C42—C43	109.45 (11)
C25—C24—H241	108.2	N40—C42—H421	109.2
C23—C24—H241	108.2	C41—C42—H421	109.2
C24—C25—H251	109.5	C43—C42—H421	109.2
C24—C25—H252	109.5	C42—C43—C44	113.48 (12)
H251—C25—H252	109.5	C42—C43—H431	108.9
C24—C25—H253	109.5	C44—C43—H431	108.9
H251—C25—H253	109.5	C42—C43—H432	108.9
H252—C25—H253	109.5	C44—C43—H432	108.9
C24—C26—H261	109.5	H431—C43—H432	107.7
C24—C26—H262	109.5	C45—C44—C43	113.88 (15)
H261—C26—H262	109.5	C45—C44—H441	108.8
C24—C26—H263	109.5	C43—C44—H441	108.8
H261—C26—H263	109.5	C45—C44—H442	108.8
H262—C26—H263	109.5	C43—C44—H442	108.8
C21—N30—C32	120.51 (11)	H441—C44—H442	107.7
C21—N30—H301	119.7	C44—C45—C46	113.8 (2)
C32—N30—H301	119.7	C44—C45—H451	108.8
O31—C31—N40	122.24 (11)	C46—C45—H451	108.8
O31—C31—C32	121.86 (10)	C44—C45—H452	108.8
N40—C31—C32	115.90 (11)	C46—C45—H452	108.8
N30—C32—C31	107.38 (9)	H451—C45—H452	107.7
N30—C32—C33	111.39 (9)	C45—C46—H461	109.5
C31—C32—C33	110.81 (10)	C45—C46—H462	109.5
N30—C32—H321	109.1	H461—C46—H462	109.5
C31—C32—H321	109.1	C45—C46—H463	109.5
C33—C32—H321	109.1	H461—C46—H463	109.5
C32—C33—C34	114.85 (10)	H462—C46—H463	109.5
C32—C33—H331	108.6		
			
O12—C10—N20—C22	0.51 (17)	N40—C31—C32—N30	112.91 (11)
C11—C10—N20—C22	−179.42 (9)	O31—C31—C32—C33	54.24 (13)
C10—N20—C22—C21	−113.52 (11)	N40—C31—C32—C33	−125.23 (10)
C10—N20—C22—C23	127.58 (11)	N30—C32—C33—C34	176.58 (9)
O21—C21—C22—N20	−53.20 (13)	C31—C32—C33—C34	57.10 (13)
N30—C21—C22—N20	128.72 (9)	C32—C33—C34—C35	59.28 (13)
O21—C21—C22—C23	65.55 (14)	C32—C33—C34—C36	−177.85 (10)
N30—C21—C22—C23	−112.52 (11)	O31—C31—N40—C42	−1.75 (17)
N20—C22—C23—C24	171.82 (10)	C32—C31—N40—C42	177.72 (10)
C21—C22—C23—C24	53.30 (13)	C31—N40—C42—C41	−63.52 (15)
C22—C23—C24—C26	64.09 (15)	C31—N40—C42—C43	176.03 (11)
C22—C23—C24—C25	−173.23 (12)	O41—C41—C42—N40	164.16 (18)
O21—C21—N30—C32	−4.26 (16)	O41—C41—C42—C43	−74.9 (2)
C22—C21—N30—C32	173.77 (9)	N40—C42—C43—C44	−63.46 (16)
C21—N30—C32—C31	−141.31 (10)	C41—C42—C43—C44	176.13 (13)
C21—N30—C32—C33	97.19 (12)	C42—C43—C44—C45	−60.78 (18)
O31—C31—C32—N30	−67.62 (13)	C43—C44—C45—C46	−173.82 (15)

### Hydrogen-bond geometry (Å, º)

**Table:**

*D*—H···*A*	*D*—H	H···*A*	*D*···*A*	*D*—H···*A*
N20—H201···O31^i^	0.88	2.05	2.897 (3)	161
N30—H301···O21^ii^	0.88	1.99	2.863 (3)	171
N40—H401···O12^i^	0.88	1.96	2.827 (3)	169

Symmetry codes: (i) −*x*+1, *y*+1/2, −*z*+1; (ii) −*x*+1, *y*−1/2, −*z*+1.
